# Identity conflicts of student affairs officers in a medical university

**DOI:** 10.1080/10872981.2023.2182216

**Published:** 2023-02-25

**Authors:** Mikio Hayashi, Raoul Breugelmans, Katsumi Nishiya

**Affiliations:** a Center for Health Professions Education, Kansai Medical University, Osaka, Japan; b Master of Medical Sciences in Medical Education, Harvard Medical School, Boston, Massachusetts, USA; c Department of English, Kansai Medical University, Osaka, Japan

**Keywords:** Student affairs officers, identity, collaboration, figured worlds theory, qualitative study

## Abstract

**Introduction:**

Collaboration between student affairs officers and the faculty is important in dealing with the recent rapid changes in medical education, and mutual understanding is essential to ensure that participants become a cohesive social group. This study explores the identity conflicts of student affairs officers in medical universities using the figured worlds theory.

**Methods:**

An exploratory qualitative case study was conducted with 24 student affairs officers at a private medical university in Japan. Data were collected through face-to-face, semi-structured interviews and analysed using thematic analysis from the perspective of a social constructivism paradigm.

**Results:**

Qualitative analysis revealed the following three themes regarding the identity conflicts of student affairs officers: differences in the perception of medical students, difficulties in building trusting relationships with the faculty, and resistance to the medical university’s traditional atmosphere. Student affairs officers tended to provide support from a student-centred perspective when interacting with medical students, while the faculty employed a teacher-centred perspective.

**Discussion:**

To promote understanding between professions, it is necessary to set aside certain professional views and welcome dialogue with other professionals with different values, while also understanding the multi-layered context of medical education, so that conflicts can be handled optimally and relationships can be professionalised for social cohesion.

## Introduction

Medical university education is complex when considering the people, situations, and systems that influence and complement each other [1]. Significant changes have occurred in medical education in recent years, including a shift toward competency-based medical education in teaching and learning, enhancement of the mentoring system, and use of new technologies, such as online learning and simulation training programmes [2–4]. Collaboration between student affairs officers (SAOs) and the faculty is important in dealing with the recent rapid changes in medical education mentioned above, where mutual understanding is essential to ensure that education is smoothly imparted and that participants become a cohesive social group [5,6]. Although the spotlight tends to focus on faculty members who actually interact with medical students during classes and in clinical situations, SAOs – who continuously and inclusively monitor their growth and development – also play an important, behind-the-scenes role [7].

There are now several types of SAOs at medical universities, and organisational members may also include physicians, counsellors, and administrative staff with no medical training. SAOs commonly handle the academic and financial affairs of all medical students, providing a supportive advisory role or responding to medical students who are experiencing various difficulties [8]. For example, SAOs support events such as entrance and graduation ceremonies, as well as student enrolment management related to withdrawals, expulsions, transfers, leaves of absence, graduation, student life guidance, and student extracurricular activities. In addition to overseeing student counselling, SAOs also serve as a liaison between the faculty council, the student affairs committee, and other education-related committees on campus. Furthermore, they work with faculty members to create lecture and practical training curricula and educational guidelines for medical students. However, there is a hierarchical relationship between SAOs and the faculty, which is difficult to observe. Hierarchy is a form of organization of power that stratifies individuals and groups based on possession of valuable social resources [9]. Hierarchy could achieve social order and promote effective coordination by setting standards for learners to aspire to and establishing shared goals and role expectations [10]. However, a hierarchical structure in which faculty members are dominant and SAOs with no medical training are subordinate is detrimental because it can inhibit interprofessional teamwork and collaboration [11]. One of the challenges in multidisciplinary collaboration is the invisible hierarchical relationship [12], and the greatest impact occurs when the hierarchy mechanism is hidden [9]. Despite the complexity of the hierarchical relationships between SAOs and faculty, there is limited research in medical education literature through the perspective of SAOs, an often-overlooked category.

While much attention has been paid to the formation of various professional identities [13–15], this study explores the identity conflicts of SAOs in medical universities using the figured worlds theory [16,17]. Figured worlds are ‘socially and culturally constructed realms of interpretation in which particular characters and actors are recognised, significance is assigned to certain acts, and particular outcomes are valued over others’ [17]. Within these figured worlds, collective stories are narrated, which form the cultural resources that SAOs draw upon to construct their identities. The authors considered it helpful to conduct a qualitative study using this theory that clarifies the relationship between self and others in unravelling the mechanisms of the hidden hierarchy that exists between SAOs and faculty.

Taking this information into account, this study intends to explore the following research question: how do SAOs with no medical training integrate their particular role into their own identities in a medical university? How do they shape the cultural resources they use to construct their own identities through their relationships and interactions with faculty members?

## Methods

### Study design

The authors used an exploratory qualitative case study methodology positioned within a constructivist paradigm [18]. A case study empirically investigates a current phenomenon occurring in a real context [19]. The strength of this approach is the consideration of various perspectives of a phenomenon, allowing for an in-depth investigation [20,21].

This study aims to clarify the hidden hierarchy that exists between SAOs and the faculty. Uncovering the complex relationships among staff members will be valuable to improve future collaboration in medical education. The authors adopted the concept of the figured worlds theory to understand how SAOs integrate this particular role into their identities, and how they shape the cultural resources they use to construct their own identities [16,17]. This study used various discourses on faculty relations to analyse SAOs’ identity conflicts using the figured worlds theory. This is based on the premise that they can use their imagination to talk about a possible future world, even when their room for initiative is limited by their assigned positions.

### Participants

The study was conducted at Kansai Medical University, a private medical university in Japan. All SAOs, excluding physicians and counsellors, were invited to participate voluntarily, as the purpose of this study was to clarify the hidden hierarchy mechanism that exists between SAOs and faculty using the figured worlds theory. The authors considered the possibility that the participation of physicians and counsellors in the clinical role might make the hierarchy less visible in this study. Overall, 24 SAOs (16 women, 8 men) agreed to participate in this study. No participant requested to discontinue their engagement after participating in the study. The median age of the participants was 36 years (IQR 21), and the median duration of continuous working years at this medical university was 5 years (IQR 4).

### Data collection

Semi-structured interviews were conducted to understand SAOs’ perspectives [22]. The first author (MH) conducted face-to-face interviews between August and November, 2021. Open-ended questions were used to clarify the participants’ perspectives on their experiences and how those experiences contributed to their situation. After confirming each SAO’s previous professional experience and the nature of their usual work, MH asked general questions about their history with the faculty members. If no difficulties were encountered, we proceeded by asking about the usual work situation. If difficulties were encountered, we asked them to describe these in detail. The key questions were: (1) ‘What kind of conflicts do you face in your daily work with the medical faculty?’ (2) ‘How do you reconcile these conflicts with your identity as a student affairs officer?’ During the interviews, the interviewer also requested background information on the SAOs, such as their main duties and reasons for choosing this job. Interviews lasting 30–60 min were conducted in Japanese and audio recorded. The recorded data were transcribed verbatim immediately after each interview, and then translated into English by the authors.

### Ethics

This study was approved by the Institutional Review Board of Kansai Medical University (2021127). The participants were informed of the study’s scope and nature, and they provided written consent prior to participation.

### Data analysis

Interview data were analysed using the thematic analysis method, which consists of generative coding and theorising to identify instances of similar concepts in the dataset [23,24]. Data analysis was conducted in two stages: the first stage used thematic analysis to continuously and iteratively analyse the interview data, while the second stage not only established themes, but also focused on the complex interaction of personal and social factors. This second-stage analysis approach allowed for greater sensitivity to the identity narratives possessed by the SAOs in light of the insights from the thematic analysis; it also helped sharpen the selection of interview data fragments. Data analysis followed an inductive approach, affected by theoretical themes from figured worlds to act as sensitising concepts [17], observing emerging conceptual categories and descriptive themes using the qualitative data analysis software package NVivo 11 (QSR International, Australia). MH and KN conducted all steps of the analysis individually, including the reading and rereading of the narratives until the researchers found the themes and categorised the data positioned within a constructivist paradigm. The researchers applied an inductive coding approach in several iterations until an agreement was achieved. For each disagreement, the authors discussed and reviewed the data until reaching consensus. The authors are currently faculty members of this medical university and are involved in the education of medical students. Given that researcher reflexivity is essential to reinforce trustworthiness, MH maintained a reflexive diary throughout the research, and the authors regularly considered their positions and assumptions during data collection and analysis [25]. Because of the assumed limitation in conducting the study of excluding SAOs with clinical roles, the interpretation of the results of the analysis was carefully reviewed to minimise arbitrary interpretations; accordingly, minor changes in direction were made when there was a possibility of emphasising SAOs’ bias.

## Results

Qualitative analysis revealed the following three themes regarding the identity conflicts of SAOs: differences in the perception of medical students, difficulties in building trusting relationships with the faculty members, and resistance to the medical university’s traditional atmosphere. Below, the authors describe the details of these themes with quotes (identified by participants’ gender and years of continuous service at Kansai Medical University).

### Differences in the perception of medical students

Through their interactions with medical students, SAOs were aware that there is a difference in the figured worlds when interacting with medical students between SAOs and the faculty. SAOs are in direct contact with medical students from the time they first enter the university and build a relationship of mutual trust through a friendly, one-on-one approach from a long-term perspective. On the contrary, faculty members tend to have a fragmented approach because they are mainly involved in their own speciality, and think about how to interact with medical students based on whether the content of their own teaching has been sufficiently conveyed to the students:

I have the impression that the image of students we see is different from that of faculty members. We see a lot of immature medical students, while faculty members see students who have become adults, including those in clinical practice, so I feel there is a big difference… I hope I can help them find a good teacher and move in the best direction possible. (Female, 17years’ experience).

Although there were individual differences among faculty members, especially when the faculty member was a clinician, the degree of interest in education was likely to differ, and it was easy to feel a sense of distance between SAOs and the faculty. Contrarily, since the SAOs lacked specialised knowledge of clinical practice, they had no choice but to defer to the faculty’s discretion, which they felt was a dilemma:

There are many clinical faculty members whose first priority is to provide medical care, so if they cannot be contacted because they are in the middle of medical care, I think it depends on what their focus is… I think it might be okay if they are highly interested in education, but I have heard that there are some faculty members who are not so interested in education… Student affairs officers do not have much knowledge of medicine, so we have to cooperate with faculty members. In addition, when it comes to clinical practice, we are in a position where we can only ask the faculty to provide education at a certain level, and I feel that administrative staff tends to be reserved. (Female, 3years’ experience).

Additionally, faculty members prioritise carrying out their duties smoothly, and are less concerned about the personal circumstances of the medical students and the relationships with students that only SAOs have. SAOs tended to provide support from a student-centred perspective when interacting with medical students, while the faculty employed a teacher-centred perspective:

In the field of education, the students are the main focus, but I think the management is not from the students’ point of view, but rather from the teachers’ point of view. For example, even in the case of clinical training, the faculty members in each department have priority. It may not be a good idea, especially since clinical faculty have their own clinics. (Male, 1 years’ experience).

I was in charge of asking the faculty to evaluate the students, but I saw glimpses of the faculty wanting to reduce their work in the medical university classes as much as possible because their main duty is medical work… I felt that their stance was different. (Female, 4 years’ experience).

### Difficulties in building trusting relationships with the faculty

SAOs thought that there was an invisible wall of tacit knowledge between the faculty’s figured world and their figured world, and that this gap might have caused discrepancies in their work. Although they understood that the exchange of opinions should be based on an equal relationship, they thought that they shared information efficiently and take arbitrary actions out of consideration for the busy schedules of faculty members. However, they felt that these responses led to their own identity conflicts as SAOs:

We rarely have a chance to share our thoughts with teachers, and even when we do, it’s only with a few teachers, so I’m wondering how we can solve this problem. If I could share my thoughts a little more, it would be an opportunity to learn more about the faculty’s thoughts. I think it will also be an opportunity for the faculty to understand the beliefs that serve as the code of conduct for student affairs officers. (Female, 6years’ experience).

Behind the reluctance to spend time discussing issues with faculty members was the SAOs’ own judgement that they should not interfere with the faculty members’ enormous clinical workload, and that they should be risk-averse because of the difficulty in repairing a broken trust relationship. Additionally, the SAOs tried to come to terms with their own identity conflicts by practicing humility in their duties and the thoughtful consideration of faculty:

While the clinical work and other difficulties of faculty members remain the same, I sometimes feel that the educational work for medical students that we ask the faculty members to do is becoming a burden as the medical education curriculum becomes more and more enormous… I believe that it would be safer if we could properly consult with them and decide on specific policies, if that is the case, but since there is not always a return for the time and effort, perhaps we should prioritise a system that gives more leeway to the faculty members. (Male, 5years’ experience).

Experienced SAOs reflected that they should not play the role of a silent intermediary based on their willingness to understand the personalities of faculty members. Based on their own successful experience of being able to express their thoughts to faculty members by building trust over time, they conveyed to their subordinates the importance of approaching faculty members without prejudice:

I believe in being able to give input to the faculty, and I have often thought about how student affairs officers should behave within the organisation… I feel that by being flexible in my thinking and reaching out to the faculty, I can get them to listen to me… I stress to my subordinates to build trust through individual cases. (Female, 27years’ experience).

### Resistance to the medical university’s traditional atmosphere

Considering the work environment of the medical university as a figured world, SAOs were aware of the traditional atmosphere of hierarchy and authority in the medical university itself, through their own work experience. Further, they felt that this traditional atmosphere may have hindered collaboration among staff members in situations that would normally be best served by multidisciplinary collaboration:

Although I believed in equal relationships between staff members in my previous job … at the medical school there is a clear hierarchical relationship based on job type and age, so I have eliminated the idea of equality… When I see young administrative staff in particular taking one or two steps back to engage with faculty members, I feel that such a culture and history has taken root. (Female, 8years’ experience).

SAOs often felt that they needed to be considerate of medical students in accordance with the uniqueness of the medical school environment. However, they felt that the authoritative situation could be alleviated by a compromise or interpretive response from the faculty to the administrative staff:

Working as a student affairs officer, I’ve come to think that listening to classes with the same members in the same classroom all the time must be a burden, considering the relationships among students. There are a lot of medical students who get mentally ill at the slightest impasse in their student lives… Although there are differences in the stance of each teacher, I feel that the high threshold among professions has been dispelled by the fact that the faculty members themselves set up meetings with the students and their parents or visit the student affairs office and care about us. (Female, 2years’ experience).

Moreover, SAOs felt that in the medical university environment, there was a tendency to stick to their traditional atmosphere, often leading to ambiguous and situationally dependent policy decisions. They felt that this situation could easily lead to conflicts in operations and delay the future development of an environment:

I feel there is a gap between the student affairs officers and the faculty, and that policies are decided based on old habits and rules, so there is little cooperation between departments and work is carried out in a stove-piped manner… It is possible in organisations other than medical universities to have strong governance by authority figures, but I sometimes feel that such an atmosphere of respect for tradition inhibits innovation in education. (Male, 3years’ experience).

## Discussion

Our case study demonstrates that SAOs felt they tended to provide support from a student-centred perspective when interacting with medical students, while the faculty employed a teacher-centred perspective. It has been shown that student-centeredness not only promotes the psychological safety of medical students and encourages their full concentration on educational activities [26], but also strengthens the relationship between medical students and faculty [27]. Therefore, the authors believe that the concept of student-centeredness as perceived by SAOs is an important perspective in the organisation of medical education and should be more respected. The limited opportunities for exchanging ideas between the faculty and SAOs were believed to have influenced the identity conflicts. Although the medical university’s traditional atmosphere, characterised by hierarchy and authority, hindered cooperation, SAOs felt they had come to terms with their own identity conflicts with humility and compassion. Our findings are valuable for medical faculties because the complexity of relationships between individuals and teams are not often emphasised in medical education [1]. The complexity of relationships is an important factor in interprofessional collaboration [28]; this study can encourage medical faculties to rethink interorganizational relationships within medical education, as well as within their own educational settings.

Based on the results of this study, the authors believe that it is necessary to discuss why SAOs reported feeling an invisible barrier of tacit knowledge between themselves and the faculty. One factor may be that a certain number of SAOs have professional experience outside of medical universities, and so may have strong awareness of the importance of collaboration among staff members. The results also suggest that the vertical environment unique to medical universities may have influenced identity conflicts. To promote understanding between professions, it is necessary to set aside certain professional views and welcome dialogue with other professionals with different values, while also understanding the multi-layered context of medical education, so that conflicts can be handled optimally and relationships remain professional for social cohesion [5,29]. It is necessary to consciously incorporate this content when implementing faculty development programmes for faculty members. Furthermore, it is believed that SAOs and faculty should collaborate to provide support to students [30], and each university should consider more comprehensive staff development programmes. One of the authors’ suggestions for specific initiatives might be to incorporate discussions, within the faculty development programme, regarding how this student-centeredness can be established in medical universities, based on the facts that student-centeredness strengthens the relationship between medical students and faculty members [27]. In addition, to improve the relationship between SAOs and faculty members, it might be helpful to include in the faculty development agenda, a discussion on how to improve their relationship, using specific case examples of relationships encountered in the educational field.

The results of this case study showed that SAOs felt they showed humility in their duties and thoughtful consideration in building relationships with faculty, and that faculty members have difficulty paying attention to the personal circumstances of medical students. The authors believe that the recent globalisation of medical education has played a role in this process. Although global trends in medical education, such as outcome-based education and professionalisation of educators, are embraced positively among Japanese medical educators [31], the vast expansion of the medical education curriculum may be exploiting the time available to faculty. As the faculty tends to focus on performing many tasks efficiently, it may be essential to create a system that allows the faculty to have more space. As a precondition to considering the relationship between SAOs and the faculty, the authors deliberate that one solution is to consider how they work together.

The figured worlds theory is generally regarded as embedded in a social context; it has been developed by considering both aspects of identity, namely that which emerges continuously through activity in cultural environments, and that which is more persistent and forms over time in those environments. As a theory of social practice, it emphasises that identity is developed and expressed through daily activities organised with others. Although several studies of medical education using the figured worlds theory have already been published [32–34], social processes such as those involved in medical education are seen to be influenced by hierarchy, power, and privilege. The interpretation of Japanese SAOs may be due, in part, to Japanese cultural characteristics, including power distance and collectivism [35]. It is difficult to deny that power relations associated with authority exist between SAOs and the faculty. The authors are concerned that this power relationship may reinforce the occupational collectivism of SAOs and faculty members, create prejudice among professions and inhibit the development of cooperative relationships. In addition, in societies with large power disparities, relationships tend to be more emotional than practical [36]. Although the faculty tend to subconsciously fixate on their thoughts, the authors considered they need to better understand the context of relationships, potential power, and value of each profession against the backdrop of the dynamic social structure in medical education. Conversely, the collective nature of the SAOs may have strengthened the identity of student affairs as a profession and led to the creation of invisible walls when considering the relationship with the faculty. Collectivism tends to be exclusionary, considers social networks as its main source of information, and prioritises group interests over individual interests. To solve this problem, as illustrated by the experiences of SAOs, staff members should approach each other without prejudice, which is important for determining action.

This study has some limitations. It was conducted at one medical university; therefore, the results may not be transferable given the importance of social context in each institution. Furthermore, the small number of participants may indicate that the conceptual framework was based on unique experiences of SAOs. Further research in other contexts is required to examine the transferability of these findings. Moreover, this study was not longitudinal. Follow-up participants would enable further observation of the development of their professional identities over time and provide more insight into how they utilised their individual experiences. In addition, further research to explain how faculty perceive their relationship with SAOs may clarify the interpretation of SAOs. Finally, by limiting the scope of this study to SAOs with no medical training background, the study may have somewhat unbalanced perspectives regarding hierarchy and roles. In other words, it may be possible that the faculty members, while being student-centred, may be struggling to spend sufficient time with SAOs due to the pressures of clinical work and time constraints. Future interviews or focus groups including SAOs with medical training backgrounds may be useful in addressing these issues.

## Conclusion

To keep up with the recent rapid changes in medical education, collaboration between SAOs and the medical faculty is crucial. Mutual understanding is essential for smooth education and social cohesion among the stakeholders. Using the figured worlds theory, this study found that multi-layered differences in perceptions between SAOs and faculty members triggered identity conflicts in medical universities. There is insufficient research focusing on the identity of SAOs who act as a bridge between medical students and faculty members in medical universities. Rather than simply highlighting differences in their perceptions, the authors believe that the results of this study should prompt all staff members to reconsider the nature of their interactions in medical education and to seek various ways to improve relationships at their institutions through faculty development programmes. The findings of this study will be useful for SAOs and faculty members to reflect on the significance of a student-centred perspective. They will also help medical faculty to consider the nature of inter-staff education and collaborative practice. Although the researchers considered that the medical faculty may tend to subconsciously fixate on their thoughts, they conceivably need to better understand the context of relationships, potential power, and value of each profession in the dynamic social structure of medical education. However, it is conceivable that faculty members may have a dilemma that prevents them from communicating adequately with SAOs due to their clinical constraints, and this study has not been able to fully clarify these facts. Future research should unravel the relationships between SAOs and medical faculty using various contexts and perspectives to find the ideal form of collaboration in medical education.

## Data Availability

The datasets generated and/or analysed during the current study are not publicly available in order to protect the originality of our work so that we can continue evaluating future participants, but are available from the corresponding author upon reasonable request.
